# Surgical timing and indications for decompressive craniectomy in malignant stroke: results from a single-center retrospective analysis

**DOI:** 10.1007/s00701-023-05817-x

**Published:** 2023-09-26

**Authors:** Silvia Hernández-Durán, Xenia Hautmann, Veit Rohde, Christian von der Brelie, Dorothee Mielke

**Affiliations:** 1 Department of Neurosurgery, Georg August University Göttingen, Robert Koch Strasse 40, 37075 Göttingen, Germany; 2 Department of Neurosurgery, Johanniter Hospital Bonn, Bonn, Nordrhein-Westfalen Germany

**Keywords:** Ischemic stroke, Decompressive craniectomy, Mortality, Timing in decompressive craniectomy

## Abstract

**Purpose:**

Acute ischemic stroke induces rapid neuronal death and time is a key factor in its treatment. Despite timely recanalization, malignant cerebral infarction can ensue, requiring decompressive surgery (DC). The ideal timing of surgery is still a matter of debate; in this study, we attempt to establish the ideal time to perform surgery in this population.

**Methods:**

We conducted a retrospective study of patients undergoing DC for stroke at our department. The indication for DC was based on drop in level of consciousness and standard imaging parameters. Patients were stratified according to the timing of DC in four groups: (a) “ultra-early” ≤12 h, (b) “early” >12≤24 h, (c) “timely” >24≤48 h, and (d) “late” >48 h. The primary endpoint of this study was in-house mortality, as a dependent variable from surgical timing. Secondary endpoint was modified Rankin scale at discharge.

**Results:**

In a cohort of 110 patients, the timing of surgery did not influence mortality or functional outcome (*p*=0.060). Patients undergoing late DC were however significantly older (*p*=0.008), and those undergoing ultra-early DC showed a trend towards a lower GCS at admission.

**Conclusions:**

Our results add to the evidence supporting an extension of the time window for DC in stroke beyond 48 h. Further criteria beyond clinical and imaging signs of herniation should be considered when selecting patients for DC after stroke to identify patients who would benefit from the procedure.

## Introduction

Acute ischemic stroke (AIS) induces rapid neuronal death, with studies quoting an average of 1.9 million neurons lost per minute of interrupted brain perfusion [19]. Thus, time is a key factor in the treatment of AIS, with current guidelines advocating for the fastest achievable onset-to-treatment time to reduce morbidity and mortality [14]. In fact, studies have shown that for every 15 min reduced in the interval between symptom onset and therapy administration, the odds of a better outcome increase [14, 20]. Hence, if treatment with adequate reperfusion is not timely performed, infarct volume increases, leading to increased disability and mortality.

Despite timely recanalization, malignant cerebral infarction (MCI) with intracranial hypertension resulting from edematous infarcted brain tissue can ensue. In these cases, decompressive craniectomy (DC) is indicated to reduce mortality [22]. Three randomized-controlled trials — DESTINY [9], HAMLET [7], and DECIMAL [23] — provided the evidence to advocate for surgery within the first 48 h after ictus in patients aged 60 years or younger, as stated in international guidelines [16]. Nevertheless, this time window has been debated, with studies showing that patients can benefit from surgical intervention beyond this time span [4]. In this study, we aim to elucidate the optimal time window to perform DC in patients with MCI.

## Methods and materials

We conducted a retrospective analysis of patients undergoing DC for MCI at our center between January 2011 and March 2019. Internal review board approval was obtained; the study was carried out in accordance with the 1964 Helsinki declaration.

### Patients

Basic demographic and clinical parameters were collected, including sex, age, Glasgow coma scale (GCS), and National Institutes for Health Stroke Scale (NIHSS) scores at presentation, comorbidities such as arterial hypertension (AHT), diabetes mellitus (DM), and hypercholesterolemia, smoking status, and positive past medical history for stroke. To better categorize stroke volume, the Alberta stroke program early CT score (ASPECTS) was applied.

### Interventions

Intravenous thrombolysis (IVtPA) was administered to patients if they presented within 3 h of ictus, in compliance with international guidelines [16, 18]. Patients underwent mechanical thrombectomy (MT) of large vessel occlusion (LVO) if they presented within 6 h from ictus, or if they met further criteria for late MT, as outlined in international guidelines [21].

### Definitions

MCI was radiologically defined as hypodensity in computed tomography (CT) and/or mismatch in CT-perfusion of more than 50–75% of the middle cerebral artery (MCA) territory, including the basal ganglia, involvement of additional vascular territories, and midline shift. Clinically, MCI was defined as progressive neurological deterioration, supposing a drop of at least two points in GCS score in non-comatose patients.

### Surgical indications and strategy

DC was indicated by a senior neurosurgeon and a senior neurologist in interdisciplinary fashion when the aforementioned criteria were met. Of note, no strict time window between symptom onset and therapy was considered when indicating DC. Exclusion criteria for surgery were (a) MCI involving the entire cerebral hemisphere (anterior, middle, and posterior cerebral artery territories); (b) bilateral AIS; (c) bilateral, fixed, dilated pupils. Hemorrhagic transformation of the MCI prior to surgery was not considered a contraindication. Age was not considered an exclusion criterion either, as premorbid performance was taken into account above chronological age alone.

### Endpoints and statistical analysis

Patients were stratified according to the timing of DC in four groups: (a) “ultra-early” ≤12 h, (b) “early” >12≤24 h, (c) “timely” >24≤48 h, and (d) “late” >48 h. Means (± standard deviations (SD)) were used to report normally distributed continues variables. Frequencies and percentages were used to report categorical and ordinal variables. The groups were compared by means of an ANOVA test for continuous variables, and through a Chi Square test for categorical ones. The primary endpoint of this study was in-house mortality, as a dependent variable from DC timing. Secondary endpoint was modified Rankin scale (mRS) at discharge. Furthermore, a multivariate regression analysis was performed to assess which factors were associated with in-house mortality. Significance was assumed at *p*<0.05. Results are reported in odds ratios (OR) and confidence intervals (CI). Statistics were performed with IBM® SPSS® v. 27.

## Results

A total of 110 patients were included, of which *n*=39, 35% perished. Mean age was 60 years (range: 18–84). Baseline characteristics are summarized in Table 1.

**Table 1:** Baseline characteristics of patients undergoing decompressive craniectomy for malignant ischemic stroke at our center

	Ultra-early DC (≤12 h)	Early DC (>12 h ≤24 h)	Timely DC (>24 h ≤48 h	Late DC (>48 h)	*p*
Total (*n*, %)	25, 23	32, 29	32, 29	21, 19	-
Demographics					
Age in years, mean (SD)	55 (12.1)	59 (8.6)	58 (15.1)	67 (9.6)	.008*
Male (*n*, %)	12, 48	23, 72	23, 72	9, 43	.049*
AHT (*n*, %)	8, 32	22, 69	25, 78	15, 71	.002*
DM2 (*n*, %)	2, 8	7, 22	8, 25	6, 29	.307
Smoker (*n*, %)	5, 20	6, 19	9, 28	4, 19	.785
Hypercholesterolemia (*n*, %)	0, 0	6, 19	8, 25	3, 14	.069
Previous stroke (*n*, %)	1, 4	5, 16	4, 12	2, 10	.555
Clinical and radiological signs					
GCS at presentation, mean (SD)	8 (4.5)	10 (3.9)	10 (4.3)	11 (4.6)	.074
Side of infarction, right (*n*, %)	13, 52	17, 53	18, 56	14, 67	.744
NIHSS at presentation, mean (range)	14 (2.8)	17 (5.0)	15 (5.5)	16 (5.6)	.755
ASPECTS score, mean (range)	3 (2.2)	2 (1.9)	2 (1.9)	3 (1.3)	.469
Length of hospitalization in days, mean (SD)	14 (9.8)	24 (13.5)	22 (10.9)	29 (26.3)	.018*
Procedures					
Intravenous thrombolysis (n, %)	11, 44	13, 41	15, 47	12, 57	.690
Mechanical thrombectomy (*n*, %)	7, 28	13, 41	14, 44	11, 52	.396

*AHT* Arterial hypertension; *ASPECTS* Alberta stroke program early CT score; *DC* decompressive craniectomy; *DM2* diabetes mellitus type 2; *GCS* Glasgow coma scale; *NIHSS* National Institutes for Health Stroke Scale; *SD* standard deviation

*Statistical significance

Most patients (*n*=76, 68%) exhibited a significantly decreased level of consciousness (GCS<13). A trend towards severe impairment of consciousness (GCS<9) was observed in patients undergoing ultra-early DC (*p*=0.074), without it reaching statistical significance. All patients exhibited large infarct volumes at presentation, with a mean ASPECTS of 3 (0–10).

No statistically significant differences were observed in thrombolytic procedures performed, with *n*=51, 46% of all patients receiving IVtPA, and *n*=45, 41% undergoing MT.

Of note, patients undergoing late DC were significantly older (mean 67 years, *p*=0.008) than those undergoing DC within 48 h after symptom onset. Furthermore, most patients undergoing DC between 12 and 48 h were males, while those undergoing ultra-early or late DC were almost equally distributed between male and female. This difference in sex distribution was statistically significant (*p*=0.049). On the other hand, patients undergoing ultra-early DC did not have AHT as a previously known comorbidity (*p*=0.002) and had significantly shorter lengths of hospitalization (*p*=0.018), with a mean of 14 days (SD 9.8).

**Fig. 1:**
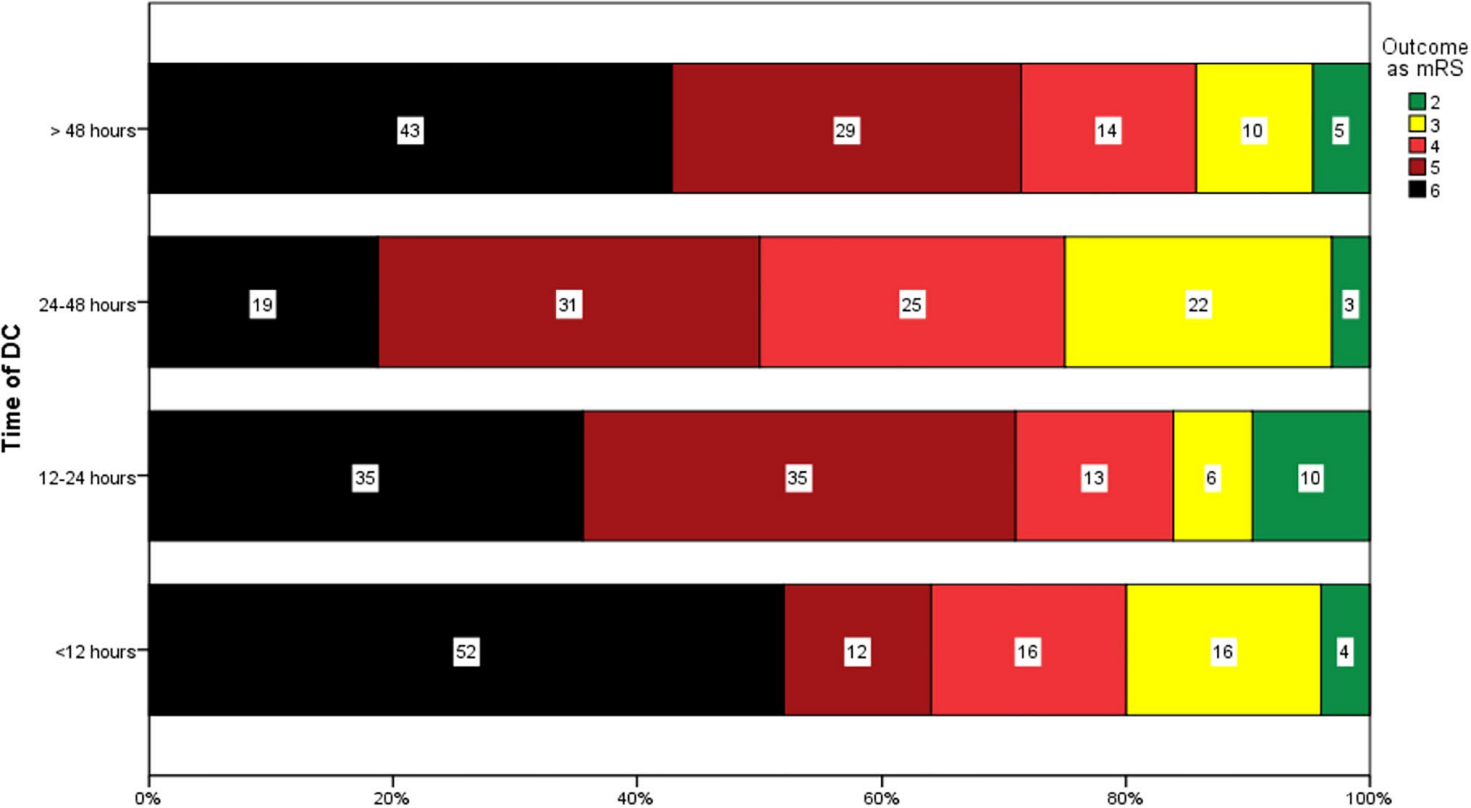
Patients’ functional outcome according to mRS at discharge after undergoing DC for MCI, stratified by timing of surgery

While no statistical significance was achieved (*p*=0.060), patients undergoing ultra-early DC and late DC had higher mortality rates of 52% and 43%, respectively. Primary and secondary outcomes are summarized in Table 2. Favorable outcomes of mRS≤3 were observed in a minority of cases, as summarized in Fig. 1. In a multivariable analysis, only previous stroke (*p*=0.044) was predictive of in-house mortality.

**Table 2:** Primary (mortality) and secondary (modified Rankin Scale) outcomes depending on the time to decompressive craniectomy

	Ultra-early DC (≤12 h)	Early DC (>12 h ≤24 h)	Timely DC (>24 h ≤48 h	Late DC (>48 h)	OR (CI)	*p*
Mortality (*n*, %)	13, 52	11, 35	6, 19	9, 43	1.33 (0.56-3.16)	.060
mRS					1.20 (0.57-3.01)	.307
1 (*n*, %)	0, 0	0, 0	0, 0	0, 0		
2 (*n*, %)	1, 4	3, 9	1, 3	1, 5		
3 (*n*, %)	4, 16	2, 6	7, 22	2, 10		
4 (*n*, %)	4, 16	4, 12	8, 25	3, 14		
5 (*n*, %)	3, 12	11, 34	10, 31	6, 29		
6 (*n*, %)	13, 52	11, 34	6, 19	9, 43		

*DC* Decompressive craniectomy; *CI* confidence interval; *OR* odds ratio; *mRS* modified Rankin scale

## Discussion

Our analysis did not show any statistical differences in mortality or functional outcome in patients undergoing DC for MCI at different time-points after symptom onset; in particular, no benefit from ultra-early DC, within the first 12 h after ictus, could be proven. Similarly, no futility could be proven in performing DC beyond 48 h after ictus. These results raise several questions about the surgical management of AIS and MCI, not only regarding the timing of DC, but also the indications therefor.

Our results add to the body of evidence in favor of extending the time window for DC in MCI. In a meta-analysis of seven studies reporting on 1508 patients, DC after 48 h of ictus was not associated with a higher risk of unfavorable outcome [4].Thus, the classical assumption that DC should be performed within 48 h after ictus should be reevaluated. While the evidence does not suffice to call for a change in the guidelines, the authors advocate for individualized decision-making when selecting patients for DC in MCI beyond 48 h after ictus.

In our cohort, patients undergoing ultra-early DC were admitted with lower GCS scores (mean=8) and comatose, as opposed to those undergoing DC later, who presented with an average GCS>8. Additionally, ASPECTS score at presentation did not differ within the groups, thus underlining the question as to whether surgical indication for DC in AIS should be based on early imaging criteria before a severe clinical deterioration ensues.

Based on our internal protocols, DC is indicated when patients suffer a drop of at least two points in GCS and/or exhibit imaging signs of herniation. In a study of 71 patients with MCI undergoing DC, Mori et al. argue that early DC, before clinical and radiological signs of herniation ensue, can improve functional outcome and reduce mortality in this patient population [12]. However, this approach has been contended in the literature. The HeADDFIRST trial attempted to identify patients with AIS who would benefit from early DC. However, of 40 patients amenable to DC, only 26 developed clinical deterioration [3], thus raising the question as to whether some patients would have undergone an unnecessary procedure with relatively high morbidity [13].

Another finding in our study was that, on average, patients undergoing late DC were significantly older. This is probably due, in part, to brain atrophy and a decreased baseline intracranial pressure (ICP) in elderly patients [15]. A previous study from our group demonstrated that elderly patients with MCI and DC have statistically significantly lower ICP values than their younger counterparts [6]. Thus, we hypothesize that these patients are better able to compensate, at least at an early stage, surges in ICP that would otherwise lead to drops in GCS and thus to a surgical indication in younger patients. This raises a further question as to whether GCS adequately reflects clinical deterioration in elderly patients. Whether elderly patients with AIS and MCI should be considered for DC has also been a matter of debate. The DESTINY II trial [10] showed that elderly patients could benefit from DC to improve mortality and outcome. On the other hand, the DECAP study showed that DC could be a life-saving measure for elderly patients as well, albeit not one positively influencing functional outcome [17].

### Future directions

Taken together, our results suggest that the indication and timing of DC in MCI warrant critical reassessment. Emerging evidence in traumatic brain injury, for example, supports the use of computed tomography perfusion (CTP) studies to better assess the level of brain impairment and guide treatment [1, 2, 8]. In stroke, CTP can be used to better identify tissue at risk for further ischemia and edema, and potentially select patients who would benefit from early DC, prior to significant clinical deterioration. Other studies have suggested that the rate at which stroke volume increases might be a good surrogate to predict the need for DC [11]. Clinically, applying the Wessex modified Richmond sedation scale (WMRSS), developed the aim of improving the early identification of MCI patients requiring DC, might aid clinicians in indicating DC, as it has been shown to outperform GCS in DC prediction [5].

On the other hand, elderly patients, due to age-related changes in brain compliance and ICP, appear to behave differently, exhibiting protracted brain swelling and, potentially, later herniation. Thus, selection criteria for these individuals should be further assessed, as clinical deterioration may be a later sign in the disease course of these individuals

### Limitations

Due to its retrospective nature, our study is subject to bias. Because of the criteria utilized at our center to indicate DC in MCI, our results can be seen as a self-fulfilling prophecy, for patients in worse neurological condition were operated on sooner. Furthermore, our study does not include long-term follow-up, which might have revealed better functional outcomes, as stroke patients are known to improve after rehabilitation. Additionally, the establishment of MT as an effective treatment for LVO has led to improvement in functional outcome of AIS patients and could have potentially influenced the outcome of subjects included in this analysis after 2015.

## Conclusion

Our study did not show any influence of the timing of surgery on mortality and functional outcome in patients undergoing DC for MCI. Our results appear to add to the evidence supporting an extension of the time window for DC in stroke beyond 48 h. Further criteria beyond clinical and imaging signs of herniation should be considered when selecting patients for DC after stroke to timely identify patients who would benefit from the procedure before herniation ensues.
